# Medical Treatment for Osteoporosis: From Molecular to Clinical Opinions

**DOI:** 10.3390/ijms20092213

**Published:** 2019-05-06

**Authors:** Li-Ru Chen, Nai-Yu Ko, Kuo-Hu Chen

**Affiliations:** 1 Department of Physical Medicine and Rehabilitation, Mackay Memorial Hospital, Taipei 10449, Taiwan; gracealex168@gmail.com (L.-R.C.); Naiyuko@gmail.com (N.-Y.K.); 2 Department of Mechanical Engineering, National Chiao-Tung University, Hsinchu 30010, Taiwan; 3 Department of Obstetrics and Gynecology, Taipei Tzu-Chi Hospital, The Buddhist Tzu-Chi Medical Foundation, Taipei 23142, Taiwan; 4 School of Medicine, Tzu-Chi University, Hualien 97004, Taiwan

**Keywords:** osteoporosis, anti-resorptive drugs, molecular pathway, genetics

## Abstract

Osteoporosis is a major concern all over the world. With aging, a gradual loss of bone mass results in osteopenia and osteoporosis. Heritable factors account for 60–80% of optimal bone mineralization. Modifiable factors, such as weight-bearing exercise, nutrition, body mass, and hormonal milieu, play an important role in the development of osteopenia and osteoporosis in adulthood. Currently, anti-resorptive agents, including estrogen, bisphosphonates, and selective estrogen receptor modulators (SERMs), are the drugs of choice for osteoporosis. Other treatments include parathyroid hormone (PTH) as well as the nutritional support of calcium and vitamin D. New treatments such as tissue-selective estrogen receptor complexes (TSECs) are currently in use too. This review, which is based on a systematic appraisal of the current literature, provides current molecular and genetic opinions on osteoporosis and its medical treatment. It offers evidence-based information to help researchers and clinicians with osteoporosis assessment. However, many issues regarding osteoporosis and its treatment remain unknown or controversial and warrant future investigation.

## 1. Overview

Although bone remodeling is a constant process, changes during menopause cause significant bone loss and increase the risk of osteoporotic fractures [1]. Anti-resorptive agents, including estrogen, bisphosphonates, and selective estrogen receptor modulators (SERMs), have been the drugs of choice for osteoporosis; new developments such as acidic oligopeptide-conjugated E2 [2] and tissue-selective estrogen receptor complexes (TSECs) [3] are currently in use too. Moreover, because bisphosphonates can cause osteonecrosis of the jaw (ONJ), new strategies, such as modifying bisphosphonates to target proteins or nanoparticles, are under research [2]. In the bone remodeling cycle, a major discovery is the important role of the receptor activator of nuclear factor kappa B ligand (RANKL) in osteoclast formation, and this has prompted the development of monoclonal antibodies that target RANKL for the treatment of osteoporosis. In addition, 27-Hydroxycholesterol was found to function as an endogenous SERM [3]. 

The genetics of osteoporosis remain to be elucidated. The past two decades of research have suggested that many factors interact with each other and that each gene exerts a small effect. Gene expression profiles suggested that SMAD4, CACNG1, and TRIM63 may have vital roles in the molecular mechanism of postmenopausal osteoporosis and that miR-331 may be a potential biomarker for postmenopausal osteoporosis, however, further studies are required [4].

**Figure 1:**
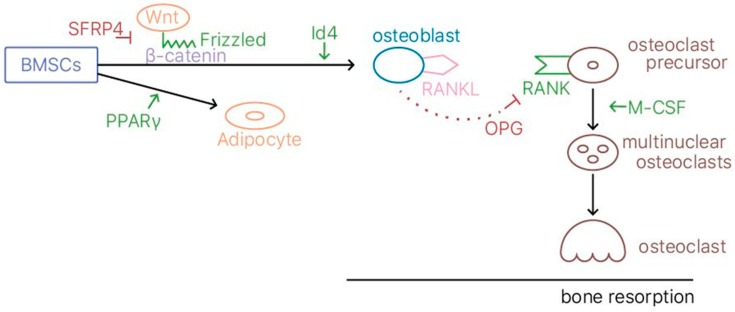
A summary of the molecular pathway underlying the action of osteoblasts, osteoclasts, and subsequent osteoporosis.

Transgenic and knockout mouse models have been employed for studying human osteoporosis, due to the similarities between human and mice genomes [5]; the ovariectomized rat model mimics osteoporosis [3,6]. Senescence-accelerated mouse prone 6 (SAMP6) mice have reduced osteoblastic bone formation, which may be due to the upregulation of the transcription factor peroxisome proliferator activator gamma (PPARγ). Moreover, the SFRP4 gene may be associated with low trabecular bone mass in SAMP6 mice by modulating the Wnt signaling pathway [7]. A summary of the molecular pathway underlying the action of osteoblasts, osteoclasts, and subsequent osteoporosis is displayed in Figure 1.

Various methods have been used to research the genetics of osteoporosis. For example, candidate gene association studies have provided strong evidence for the actions of vitamin D receptors (VDR), ERα, COL1A1, the OPG-RANKL system, and LDL receptor–related proteins (LRP5), and Id4 [5,8,9,10]. In addition, genome-wide linkage studies have revealed various quantitative trait loci (QTLs) [5], and genome-wide association studies provide new insights by using SNPs and CNVs. [5]. Functional genomic studies using DNA microarrays have found factors in CCR3, such as HDC and GCR, in the bone marrow [9]. Gene expression microarray studies and proteonomics have also increased our understanding of osteoporosis.

Finally, a cross-linked core molecular network structure could serve as a unified molecular mechanism for different phenotypes of osteoblasts [6]. Apart from genetic factors, hypovitaminosis D is also known to increase the risk of osteoporosis [11]. A clear understanding of the pathogenesis of osteoporosis can help provide direct therapeutic targets for new drugs.

Osteoporosis markedly increases the risk of skeletal fractures as well as morbidity and mortality. Effective fracture prevention by reducing the loss of bone mass is the primary treatment goal for people with osteoporosis. In addition to lifestyle changes, nutrition changes, and fall prevention, therapeutic choices such as pharmacologic agents, exercise, and physical therapy modalities can be considered to treat osteoporosis or prevent further osteoporotic fracture. Each choice or combination of choices can offer benefits for people with osteoporosis. The selected treatment should be started after a detailed discussion with patients.

## 2. Molecular Opinion

### 2.1. Bone Remodeling

On a molecular level, there are four stages of bone remodeling: (1) activation of osteoclast precursors, which mature into multinuclear osteoclasts under the direction of cytokines and hormones; (2) resorption of bone by osteoclasts, which forms a resorption cavity; (3) reversal of the resorption signal; and (4) formation of a new bone that fills up the resorption cavity [1]. During menopause, the number of resorption cavities increases. However, because bone formation does not increase accordingly, the cavities are not filled in completely with new bones, which results in a permanent loss of bone mass and an increase in the risk of osteoporotic fractures [1].

Although osteoclasts are the main targets of osteoporosis therapy, osteoblasts have been established recently as potential targets. Osteoblasts are derived from bone marrow stromal cells (BMSCs), which are self-renewing in vivo. BMSCs can differentiate into osteoblasts, chondrocytes, and adipocytes [7]. Postmenopausal osteoporosis is often accompanied by increased bone marrow adipose tissue. These progenitors are believed to form adipocytes instead of osteoblasts. Thus, we can obtain a theoretical basis for the functional modulation of BMSCs in regenerative therapy for osteoporosis [7].

Osteoclasts are large multinucleated giant cells that resorb the bone matrix, ensuring development and continuous remodeling of the skeleton and the bone marrow haematopoietic niche [12]. Defective osteoclast activity leads to osteopetrosis and bone marrow failure, whereas excess activity can contribute to bone loss and osteoporosis [12]. The osteoclasts originate from embryonic erythro-myeloid progenitors and possess an elaborate ensemble of intracellular organelles through which solutes, proteins, and other macromolecules are trafficked to their target destinations via membrane-bound intermediaries [13]. Of these organelles, the ruffled border (RB) is most characteristic, functioning as the osteoclasts’ secretory apparatus. This highly convoluted organelle is classically considered to be formed by the targeted fusion of acidic vesicles with the bone-facing plasma membrane [13]. New evidence discloses that the RB is far more complex than previously envisaged, possessing discrete subdomains that are serviced by several intersecting endocytic, secretory, transcytotic, and autophagic pathways [13]. Among these distinct vesicle transport routes, small Rab GTPases, their binding partners and members of the endocytic sorting nexin family have emerged as critical regulators [13].

Wnt signaling is a critical regulator of bone homeostasis by stimulating osteoblast differentiation and function and inhibiting osteoclast differentiation, mostly via indirect mechanisms through osteoblasts [14] (TRACP-5b TRACP-5b). It can be classified into the noncanonical (β-catenin-independent) and canonical (β-catenin dependent) signaling pathways [15]. In the former, after activation of frizzled (FZD) by noncanonical Wnt ligands, the signal which is mediated by G-proteins and c-Jun N-terminal kinase (JNK) activates Jun and the Sp1 transcription factor to express the target genes such as RANK in osteoclasts and runt-related transcription factor 2 (Runx2) in osteoblasts. The signal mediated by G-proteins activates calcium calmodulin-mediated kinase II (CaMKII) and calcineurin by increasing the amount of Ca2+ in the cytoplasm. CaMKII leads to the suppression of the adipocyte differentiation of mesenchymal stem cells, and calcineurin activates the nuclear factor of activated T cells (NFAT), an activator of bone formation and bone resorption. In the latter, activation of FZD by canonical Wnt ligands is mediated by the inhibition of GSK3 and prevents β-catenin from degradation. β-catenin accumulates in the cytoplasm and engages nuclear area to the expression of bone formation genes such as OPG and RANKL [15].

Because Wnt signaling is a crucial developmental pathway, several inhibitory safeguards exist to restrict its activity. Dickkopf-1 (Dkk1) is an important inhibitor of Wnt signaling, which binds to the Wnt co-receptors LRP5/6 and thereby blocks further interactions with Wnt ligands [14]. Recently, Colditz et al. has found that, as a negative regulator of bone formation and bone mass, Dkk1 is deregulated in bone loss induced by arthritis and glucocorticoid (GC) exposure [14]. Dkk-1 deficiency in osteolineage cells protects against GC-induced bone loss, whereas it had only minor effects on arthritis. Therefore, Dkk1 may be a promising therapeutic target, especially for bone diseases in which the inhibition of bone formation represents the predominant mechanism [14].

Two independent studies reported the identification of mouse RANKL on activated T cells and of a ligand for OPG on a murine bone marrow-derived stromal cell line. Accumulating data indicated RANKL and RANK not only as essential players for the development and activation of osteoclasts, but also for the correct differentiation of medullary thymic epithelial cells (mTECs), which act as mediators of the central tolerance process by which self-reactive T cells are eliminated while regulatory T cells are generated [16]. Considering the RANKL-RANK multi-task function, an antibody targeting this pathway, denosumab, is now commonly used in the therapy of bone loss diseases, including chronic inflammatory bone disorders and osteolytic bone metastases. Furthermore, preclinical data support the therapeutic application of denosumab in the framework of a broader spectrum of tumors [16].

Theoretically, osteoporosis treatment can target any of the four stages, however, current therapy focuses mainly on anti-resorptive agents, such as estrogen, bisphosphonates, and SERMs. Because these agents are either suboptimal or cause significant side effects, current research is attempting to develop new drugs or modify existing drugs. This review includes the literature on transgenic and knockout mouse models [5,7], a hypothesis of a cross-linked core molecular network structure [6], in silico analyses [4], candidate gene association studies, genome-wide linkage and association studies, functional genomic studies, gene expression microarray studies, and proteomics [5].

### 2.2. Animal Model

Transgenic and knockout mouse models are used for osteoporosis research due to the similarities between human and mice genomes [5,7]. Moreover, the ovariectomized rat model is similar to osteoporosis in the sense that it induces a decline of estrogen production, which results in increased bone resorption, declined bone mass, and consequently an increased risk for fragility fractures [7]. However, care should be taken when generalizing these results. SAMP6 mice, in particular, exhibit a decrease in the density of the vertebral trabecular bone. The decrease progresses faster caudally than cranially within the spine, which is similar to the situation for humans. Thus, the SAMP6 model could be compatible with human senile osteoporosis [7].

### 2.3. Estrogen and SERMs

Estrogen is perhaps the most direct and effective approach of preventing and treating osteoporosis because it inhibits osteoclastic bone resorption [7]. Osteoclasts require activation by two cytokines, namely M-CSF and RANKL, which are produced by BMSCs and osteoblasts, respectively [1]. Estrogen suppresses osteoclasts by reducing the expression of RANKL in marrow cells and increases OPG secretion by osteoblasts, which inactivates RANK [1]. However, unopposed estrogen can increase the risk of breast and uterine cancers, deep vein thrombosis, and stroke [3].

Therefore, researchers are trying to modify estrogens. Stapleton et al. reported that when estradiol (E2) was conjugated with an acidic oligopeptide, it could be selectively delivered to bone lesions. Pharmacological analysis indicated that the administration of oligopeptide-conjugated E2 in ovariectomized mice improved BMD without increasing the uterine weight, which indicates that this method effectively delivers the drug into the bone lesion while reducing unwanted side effects [2].

SERMs were found to demonstrate selectivity toward estrogen receptors (ERs) in the bone, thereby reducing side effects, however, they lack the efficacy of traditional estrogen [3]. SERMs are generally influenced by their binding affinity for ERα and ERβ and the effect of the bound ligand on the ER structure [3], however, the precise mode of action of each SERM remains unknown. One endogenous compound—27-hydroxycholesterol (27HC)—has been found to bind to and modulate the activity of ERs in vivo and to behave like an SERM. In mice, 27HC behaves as an ER antagonist and reduces the protective effects of estradiol. However, in cellular models of ER-positive breast cancer, 27HC acts a partial ER agonist. In ovariectomized mice with elevated 27HC levels, a dramatic loss of bone was observed [3]. Further research on 27HC could lead to the development of new drugs.

### 2.4. TSECs

TSECs are a new class of drugs that combine an SERM and estrogen. They have been shown to effectively treat symptoms associated with menopause and prevent bone loss, without negatively impacting the breast or uterus. Their mechanism of action is difficult to delineate, as both components of the TSEC compete for the same ligand-binding pocket on the ER [3]. However, differences are likely to exist in the pharmacodynamics and pharmacokinetics of the constituents [3]. These combinations should also have a significant influence on the development of osteoporosis drugs in the future.

### 2.5. Biphosphonates

Bisphosphonates bind to hard bone through their affinity for hydroxyapatite. Furthermore, they inhibit bone resorption and promote bone formation through osteoblast differentiation [2]. However, they can cause gastrointestinal problems, such as difficulty swallowing, esophageal inflammation, gastric ulcers, osteonecrosis of the jaw (ONJ), atrial fibrillation, and musculoskeletal pain [2]. Stapleton et al. reviewed strategies to modify bisphosphonates for targeting proteins or nanoparticles and found that, when alendronate-modified proteins were used in conjunction with polyethylene glycol (PEG) modification, an increased quantity of proteins was targeted to the bone and the plasma retention time increased [2]. Nanoparticles have also gained favor due to their small size and ability to reach targets without inducing an immune response [2]. However, this research is still in its initial stages.

### 2.6. Gene Expression

Azuma et al. found that SAMP6 mice had reduced osteoblastic bone formation and upregulation of PPARγ expression, which also corresponded with increased adipogenesis. By contrast, PPARγ-deficient ES cells spontaneously differentiate into osteoblasts and not adipocytes [7]. PPARs are members of a nuclear receptor family that form a complex with the retinoid X receptor (RXR) and function as transcription factors to regulate the expression of genes. PPARs are involved in major metabolic processes as well as cell proliferation, differentiation, and survival [6]. Due to its likely role in regulating the direction of BMSC lineage, PPARγ is regarded as a promising target for osteoporosis therapy, however, its precise mechanism remains unknown.

Another gene mentioned in the study of Azuma et al. is SFRP4, which inhibits the Wnt signaling pathway and causes low trabecular bone mass in SAMP6 mice [7]. SFRP4 polymorphism may be one of the genetic factors affecting BMD. The SFRP family contains a cysteine-rich domain, which is homologous to the putative Wnt-binding site of Frizzled proteins. SFRPs that modulate Wnt signaling are involved in bone formation [7]. SFRP4 binds to Wnt ligands and prevents their binding to the Frizzled receptor, thus inhibiting intracellular Wnt signals in the bone [7]. Wnt/β-catenin signaling then stimulates osteoblast generation by steering multipotential BMSCs toward the osteoblast lineage and suppressing the commitment to the adipogenic lineage. The Wnt signaling pathway plays a vital role in osteoblast and BMSC proliferation and differentiation in vitro. The down-regulation of this pathway results in reduced bone mass in SAMP6 mice. β-catenin is a key protein in the Wnt/β-catenin signaling transduction and promotes bone formation [7]. LRP5 mutation affects the Wnt/β-Catenin pathway, which leads to either high bone mass or osteoporosis [1].

In mammals, PiT1/SLC20A1 and PiT2/SLC20A2 are the major phosphate (Pi) transporters expressed, and they are thought to have a pivotal role in providing Pi for mineralization. SLC20A2, encoding the phosphate transporter PiT2, is an important genetic determinant of bone quality and strength. Recently, Beck-Cormier et al. identified impaired bone quality and strength in SLC20Aa2–/– mice lacking the phosphate transporter SLC20A2 [17]. Juveniles had abnormal endochondral and intramembranous ossification, decreased mineral accrual, and short stature. Adults exhibited only small reductions in bone mass and mineralization, but had a profound impairment in bone strength. Their studies identify SLC20Aa2 as a physiological regulator of tissue mineralization and highlight its critical role in the determination of bone quality and strength [17].

By estimating heel quantitative ultrasounds in 426,824 individuals, Morris et al. assessed genetic determinants of BMD and identified 518 genome-wide significant loci, which explained 20% of its variance [18]. They then performed rapid-throughput skeletal phenotyping of 126 knockout mice with disruptions in predicted target genes and found an increased abnormal skeletal phenotype frequency compared to 526 unselected lines (*p* < 0.0001). In-depth analysis of one gene, *DAAM2*, showed a disproportionate decrease in bone strength relative to mineralization [18]. This genetic atlas provides evidence linking associated SNPs to causal genes, offers new insight into osteoporosis pathophysiology, and highlights opportunities for drug development.

Osteoporosis is a multifactorial disease in which genetic factors and epigenetic modifications play a major role. DNA methylation is known for gene silencing and its effect on bone morphogenetic protein (BMP) 2 promoter has been studied to understand its regulatory activity in osteoporosis pathogenicity. In a study conducted by Raje and Ashma, CpG methylation in the BMP2 promoter was analyzed by performing bisulfate specific PCR on the gDNA samples of osteoporotic and healthy individuals [19]. Functional and gene expression analysis of this methylated site suggests reduced transcriptional activity of the BMP2 promoter as well as decreased gene expression in the disease condition. Given BMP2 is a central signaling molecule, aberrant methylation in the promoter region may result in down regulation of the osteoblast markers involved in bone formation [19].

Genetic disorders with skeletal fragility provide insight into metabolic pathways contributing to bone strength. In a recent study, Pekkinen et al. evaluated six families with rare skeletal phenotypes and osteoporosis by next-generation sequencing. All families were identified with a heterozygous variant in the sphingomyelin synthase 2 gene (SGMS2), a gene prominently expressed in cortical bones and encoding the plasma membrane-resident SMS2 [20]. Four families shared the same nonsense variant, c.148C>T (*p*.Arg50 *), whereas the other families with more severe clinical manifestations had a missense variant, c.185T>G (*p*.Ile62Ser) or c.191T>G (*p*.Met64Arg). Bone biopsies for all showed markedly altered bone material characteristics, including defective bone mineralization. SGMS2 pathogenic variants underlie a spectrum of skeletal conditions, ranging from isolated osteoporosis to complex skeletal dysplasia, suggesting a critical role for plasma membrane-bound sphingomyelin metabolism in skeletal homeostasis [20].

Osteoporosis and fracture risk are common complex diseases, caused by the interaction of numerous disease susceptibility genes and environmental factors. With the advances in genomic technologies, large-scale genome-wide association studies (GWAS) have been performed which have broadened our understanding of the genetic architecture and biological mechanisms of complex disease. Currently, more than 90 loci have been found to be associated with BMD, over 500 loci with heel estimated BMD, and several others with other less widely available bone parameters such as bone geometry, shape, and microarchitecture [21]. Notably, several of the pathways identified by the GWAS efforts correspond to pathways that are currently targeted for the treatment of osteoporosis. Overall, tremendous progress in the field of the genetics of osteoporosis has been achieved with the discovery of WNT16, EN1, DAAM2, and GPC6, among others [21].

GWAS have repeatedly identified genetic variants associated with BMD and osteoporotic fracture in non-coding regions of C7ORF76, a poorly studied gene of unknown function. In a recent study, Roca-Ayats et al. re-sequenced the genomic region in two extreme BMD groups to search for functionally relevant variants. Of eight selected variants tested for association in the complete cohort, two (rs4342521 and rs10085588) were found to be significantly associated with lumbar spine BMD and associated with osteoporotic fracture [22]. Further analyses of these two SNPs, together with SNP rs4727338, disclosed a statistically significant influence on the expression of the proximal neighboring gene SLC25A13. They analyzed the functionality of the C7ORF76 genomic region and provided functional regulatory evidence for the rs10085588, which may be a causal SNP within the 7q21.3 GWAS signal for osteoporosis [22].

The gene inhibitor of DNA binding 4 (Id4), which is part of the bHLH superfamily, also promotes osteoblast differentiation [10]. The bHLH superfamily transcription factors are upregulated in the early phase of osteoblast differentiation [10]. Studies using Id4-knockout mice have indicated that Id4 knockdown suppresses the osteoblast differentiation of MSCs and increases adipocyte differentiation, whereas Id4 overexpression promotes osteoblast differentiation and decreases lipid accumulation. Because it can direct osteogenic and adipogenic cell fate, Id4 is another likely target for preventing (or delaying the onset of) senile osteoporosis [10]. Figure 1 displays the molecular signaling pathways underlying the actions of osteoblasts, osteoclasts, and subsequent osteoporosis.

Thus, molecular signaling pathways for osteoporosis are extensive, and the current method for studying osteoporosis is often based on assigning a pro-osteoporosis or an anti-osteoporosis role for each risk factor. However, this method has led to contradictions, because it fails to account for multiple effects simultaneously [6]. Yuan et al. proposed a cross-linked core molecular network structure, which could serve as a unified molecular mechanism for different phenotypes of osteoblasts [6]. A hub-like interaction network was constructed from cross-talks among estrogens, glucocorticoids, retinoic acids, PPAR, VDR, and calcium-signaling pathways. This network provides the basis for further research and reminds us of the complex interactions between each risk factor.

VDR in particular has been strongly associated with osteoporosis. Vitamin D is a prohormone that regulates calcium and phosphate balance and bone structure. Hypovitaminosis D increases the risk of osteoporosis [11]. Calcium and vitamin D supplements have been used for the prophylaxis of osteoporosis [6]. The main molecular action of 1,25(OH)2D is to initiate or suppress gene transcription by binding to VDR [6]. It then regulates gene transcription by binding VDR to RXR. VDR/RXR is a heterodimer complex involved in regulating many key transcription pathways, the most important being the pathways activated by insulin, transforming growth factor-beta1, Wnt, and various cytokines [11].

A bioinformatics method proposed by Liu et al. attempts to analyze several genes simultaneously. In their study, 482 differentially expressed genes (DEGs) were screened in patients with postmenopausal osteoporosis. Potential transcription factors (TFs) and microRNAs were identified, along with functional modules in the protein–protein interaction (PPI) network of the DEGs [4]. First, SMAD4 expression was found to be upregulated in postmenopausal osteoporosis. Moreover, several microRNAs were implicated. The miR-148a gene promoted osteoclastogenesis, whereas miR-133a and miR-422a were upregulated with low BMD in human osteoclast precursors. Another DEG, voltage-dependent calcium channel γ1 (CACNG1), may be associated with the muscle contraction pathway. Because patients with PO often have hypercalciuria with normal blood Ca^2+^ levels, CACNG1 is thought to be involved in the mechanisms of postmenopausal osteoporosis through Ca^2+^ regulation during muscle contraction. TRIM63, which is known as muscle-specific ring finger protein 1, was identified as a hub of the functional module of the PPI network. Its overexpression may increase the expression of alkaline phosphatase, which is an osteoblastic differentiation marker gene. The increased expression of alkaline phosphatase results in reduced proliferation. TRIM63 is involved in both osteoblastic bone formation and osteoclastic bone resorption. Although the results of this study are promising, supporting experimental data are required [4].

Finally, we provide a summary of comprehensive literature reviews regarding the genetics of osteoporosis [5,8,9]. From candidate gene association studies, research discovered strong evidence for the actions of VDR, ERα, COL1A1, and LRP5 [5,8,9]. Genome-wide linkage studies have revealed various QTLs. However, research has still been unable to account for genetic heterogeneity, due to small sample sizes and different ethnicities [5]. On the other hand, genome-wide association studies provide new insight using SNPs and CNVs. Although SNPs identified five genomic regions near RANKL, OPF, and ESR2, there is only 1–4% variation for each SNP, however, at the very least, the SNPs can help identify targets. One deletion in a CNV, UGT2B17 in 4q13.2, has been shown to increase testosterone and estradiol levels [5]. Functional genomic studies using DNA microarrays have found factors in CCR3, such as HDC and GCR, in the bone marrow. HDC and GCR are upregulated in people with low BMD [9]. Gene expression microarray studies (at the mRNA level) using cultured cell lines are also invaluable for understanding the pathogenesis of osteoporosis. There is now increased understanding of the regulation of osteoblast and osteoclast activity, the proliferation and differentiation of MSCs, gene expression in healthy versus diseased tissues, the effects of therapeutic agents on the healing of fractures, and the endocrine regulation of bone remodeling [5]. Recent advances in proteomics studies have recognized the importance of epigenetics and alternative splicing. Fourteen proteins have been identified during osteoporosis research involved in glycolysis, signaling, redox, and the cartilage matrix. Although still in its initial stages, proteomics can provide us with additional knowledge [5]. Readers can refer to aforementioned reviews for a detailed analysis.

## 3. Clinical Opinion

### 3.1. Introduction

Osteoporosis is a major concern all over the world. Both sexes are affected by osteoporosis, however, an increased prevalence of the disease is observed in menopausal women. Osteoporosis markedly increases the risk of skeletal fractures, as well as morbidity and mortality [23,24]. Effective fracture prevention achieved with reduced loss of bone mass can have a major influence on women’s morbidity and, to a lesser extent, mortality.

The diagnostic difference between osteopenia and osteoporosis is based on the level of BMD. The World Health Organization (WHO) has set the definitions of osteopenia and osteoporosis according to the BMD at the spine, hip, or forearm, measured by dual-energy X-ray absorptiometry (DXA) devices [25]. According to the WHO criteria, the BMD for osteopenia is between 1.0 and 2.5 SD below that of a “young normal” adult (T score between −1.0 and −2.5), and the T score for osteoporosis is −2.5 or lower. Clinically, the occurrences of low-energy vertebral and hip fractures should also indicate osteoporosis.

Some people may have a congenitally low BMD. Childhood and adolescence are important stages for optimal bone formation and the prevention of osteoporosis in older age. Although heritable factors account for 60–80% of optimal bone mineralization, modifiable factors, such as weight-bearing exercise, nutrition, body mass, and hormonal milieu, play important roles in the development of osteopenia and osteoporosis in adulthood.

The main goal of screening for and treating osteoporosis is to prevent the development of subsequent osteoporotic fractures. Effective fracture prevention would considerably affect women’s morbidity and mortality. BMD changes with treatment may provide a suitable prediction of the anti-fracture efficacy of a therapeutic agent.

Adequate calcium and vitamin D supplementation and exercise are some of the cornerstones of osteoporosis prevention and are necessary adjuncts during treatment with pharmacological agents. Osteoporotic medications are effective only if the patient is supplied with adequate vitamin D and calcium. A variety of new therapeutic agents are now available for the treatment of osteoporosis. This review is based on a systematic appraisal of the current literature. It offers evidence-based information with the aim of providing clinicians with an assessment of the osteoporosis treatment effect. Although most therapeutic agents have been proven to significantly reduce the occurrence of vertebral fractures, controversy remains regarding the level of evidence related to their nonvertebral or hip anti-fracture effect. A summary of current medical treatments for osteoporosis is provided in the following subsections.

### 3.2. Pharmacological Treatment

Numerous therapeutic agents, which increase bone mass by inhibiting resorption (anti-resorptive drugs) or stimulating bone formation (anabolic medications), have demonstrated the effects of increased BMD and decreased risk of skeletal fractures [26]. Treatment of osteopenia or osteoporosis is primarily based on anti-resorptive drugs (bisphosphonates, estrogens, and SERMs). On the other hand, parathyroid hormone (PTH) given at a low dosage intermittently can act as an anabolic agent to stimulate bone formation.

#### 3.2.1. Estrogen Therapy (ET)/Hormone Replacement Therapy (HRT)

ET/HRT has been used for the relief of postmenopausal symptoms. Menopause-associated symptoms, including hot flushes, sweating, insomnia, mood changes, and vaginal dryness, can be improved effectively with HRT. Moreover, HRT increases BMD and reduces the incidence of osteoporotic fractures. However, its use has been challenged since Women’s Health Initiative (WHI) studies disclosed that prolonged use of HRT, especially in elderly women, increases the risk of breast cancer, thromboembolic disease, and cerebrovascular accidents, although the risk of colorectal cancer is decreased.

Currently, ET/HRT is approved by the FDA for the prevention of osteoporosis and relief of the vasomotor symptoms and vulvovaginal atrophy associated with menopause. Estrogen-only therapy is suggested for women who have undergone a hysterectomy and require therapy with hormones, whereas combined progestin and estrogen is given to women with an intact uterus to prevent the adverse effects of unopposed estrogen exposure on the endometrium. Endogenous estrogen helps in maintaining bone mass, and its deficiency after menopause has been associated with rapid bone loss. Convincing evidence shows that estrogen stimulates the apoptosis of osteoclasts and suppresses the apoptosis of osteoblasts and osteocytes by acting via ERα. Bagger et al. [27] evaluated 347 healthy postmenopausal women with normal bone mass who had earlier participated in placebo-controlled HRT trials. They reported that, compared with the placebo-treated women, the HRT-treated women had a significantly reduced risk of osteoporotic fractures (RR: 0.48; 95% CI: 0.26–0.88) [27]. According to the report of the WHI, five years of HRT with conjugated equine estrogen (0.625 mg) and medroxyprogesterone (2.5 mg) (Prempro^®^) reduced the risk of clinical vertebral and hip fractures by 34% and other osteoporotic fractures by 23% [28], however, this treatment increased the risks of myocardial infarction, stroke, invasive breast cancer, pulmonary emboli, and deep vein phlebitis [29,30]. In the estrogen-only arm of the WHI, no increase was noted in the incidence of breast cancer over 7.1 years of treatment. Moreover, subsequent analysis of these data revealed no increased risk for cardiovascular disease if none was present when HRT was initiated. However, because other studies have reported that the prolonged use of HRT, especially in elderly women, increases the risk of breast cancer, thromboembolic disease, and cerebrovascular accidents [30], HRT is no longer recommended as first-line therapy for osteoporosis. ET/HRT should be given in the lowest effective doses for the shortest duration to meet the treatment goals. Before using ET/HRT solely for the prevention of osteoporosis, the FDA recommends that approved non-estrogen treatments should first be carefully considered [29].

Another concern is the use of tibolone (Livial^®^, 2.5 mg tablet), which is a new HRT regimen consisting of a synthetic steroid hormone broken down by the body’s metabolism into three compounds that act similar to naturally-produced estrogen, progesterone, and testosterone [31]. These metabolites are tissue-specific. Once metabolized, tibolone has estrogenic effects on bone, vaginal, and breast tissues, progesterone effects on the endometrium, and androgenic effects (such as testosterone) on the brain and liver. These effects help restore the hormonal balance to relieve menopausal symptoms, such as vaginal atrophy, loss of bone density, and osteoporosis, as well as other symptoms, such as hot flushes and decreased libido. Tibolone also has other beneficial effects, such as lowering cholesterol levels, which tend to increase after menopause.

Cummings et al. [32] conducted an RCT in elderly women with osteoporosis of the hip or spine or osteopenia, and used radiologic evidence of a vertebral fracture to evaluate the effect of tibolone (1.25 mg/day, i.e., half the conventional dose). After a median time of 34 months of treatment, they found that the tibolone group had a decreased risk of vertebral fractures (RR: 0.55; 95% CI: 0.41–0.74; *p* < 0.001) and nonvertebral fractures (RR: 0.74; 95% CI: 0.58–0.93; *p* = 0.01) compared with the placebo group. Interestingly, the tibolone group also had a decreased risk of invasive breast cancer (RR: 0.32; 95% CI: 0.13–0.80; *p* = 0.02) and colon cancer (RR: 0.31; 95% CI: 0.10–0.96; *p* = 0.04). However, because the tibolone group had an increased risk of stroke (RR: 2.19; 95% CI: 1.14–4.23; *p* = 0.02), the study was stopped prematurely. Current opinion suggests that tibolone should not be viewed as first-line therapy for osteoporosis.

Regarding the effect of phytoestrogens on osteoporosis, only short-term randomized trials are available. Most of these trials evaluated either bone turnover or the modification of the bone mass, which revealed inconsistent results. With the exception of a prospective trial assessing the effects of ipriflavone on osteoporotic fractures, which concluded in the absence of a significant effect [33], limited randomized trials have evaluated the fracture efficacy of phytoestrogens [34,35].

In conclusion, ET/HRT should be considered in postmenopausal women with osteoporosis only when the benefits outweigh the risks. The details of ET/HRT should be explained to women who are considering this treatment. Although ET/HRT significantly decreases bone loss and the risk of osteoporotic fractures, its main indication in postmenopausal women remains the relief of menopausal symptoms.

#### 3.2.2. Bisphosphonates

Bisphosphonates, including alendronate, ibandronate, risedronate, and zoledronic acid (ZA), are now available for the treatment of osteoporosis. Their binding affinity and anti-resorptive potency differ among different compounds. The binding affinities of bisphosphonates are ranked as follows: zoledronate > alendronate > ibandronate > risedronate. Higher-affinity bisphosphonates bind tightly to the bone surface, however, they spread through bone slowly and have limited access to the osteocyte network. By contrast, lower-affinity agents are distributed widely throughout the bone and also have a shorter residence time in the bone than higher-affinity agents do if treatment is stopped [36,37].

The mechanism of action of bisphosphonates involves osteoclast inhibition, which reduces bone mass resorption and increases the bone density. Bisphosphonates act by inhibiting a key enzyme necessary for osteoclasts to function and survive. Thus, they induce apoptosis of osteoclasts and reduce their number on bone remodeling surfaces [38]. In comparison to placebos, all bisphosphonates improve bone density and reduce the occurrence of osteoporosis-induced fractures in both men and women. They have been recognized to be a cost-effective option for the prevention and treatment of osteoporosis in postmenopausal women with low BMD or prevalent vertebral fractures [39,40,41].

Alendronate (Fosamax^®^, for prevention, 5 mg daily or 35 mg weekly tablets, and for treatment, 10 mg daily or 70 mg weekly tablet or 70 mg weekly tablet with 2.800 IU or 5.600 IU of vitamin D3), one of the most popular bisphosphonates, is approved by the FDA for the prevention and treatment of postmenopausal osteoporosis. Alendronate is also approved for increasing bone mass in men with osteoporosis and for the treatment of osteoporosis in men and women taking glucocorticoids. It may increase BMD of both the hip and spine by 1–2% and 2–4% per year, respectively, and may decrease the risk of fractures of the hip and spine by 51% and 63%, respectively [29,36]. Over three years, it reduces the incidence of spine and hip fractures by approximately 50% in patients with a prior vertebral fracture and reduces the incidence of vertebral fractures by approximately 48% in patients without a prior vertebral fracture [29].

Since the prolonged use of bisphosphonates (BP) may lead to adverse events, some recommendations suggest consideration of a BP holiday in individuals taking long-term BP therapy, who are not at high risk of fracture. In a study, Boskey et al. hypothesized that a BP holiday of five years would cause no major bone compositional changes. They analyzed the 31 available biopsies from two groups of post-menopausal women, a continuously BP-treated group receiving alendronate for 10 years and a BP-discontinued group, using alendronate for five years without antiresorptive medication during the following five years [42]. Key parameters, such as mineral-to-matrix ratio, carbonate-to-phosphate ratio, acid phosphate substitution, and collagen cross-link ratio, were similar for both groups in age-adjusted models. They concluded that the discontinuation of alendronate for five years did not affect key bone parameters, implying that discontinuation a five-year BP holiday would have little impact on bone composition [42].

Because the comparative effectiveness of continuing or discontinuing long-term alendronate on fractures is unknown, Wright et al. conducted a six-month pilot study among current long-term alendronate users (women aged ≥65 with ≥three-year use) to evaluate outcomes one and six months after randomization, including adherence and bisphosphonate-associated adverse events [43]. For 27 participants (13 to continue alendronate, and 14 to discontinue alendronate), 22% of participants did not adhere to their randomization assignment: 30.8% in the continuation arm and 14.3% in the discontinuation arm. No fractures or adverse events were reported [43].

Romosozumab is a monoclonal antibody that binds to and inhibits sclerostin, increases bone formation, and decreases bone resorption. On 9 April 2019, the FDA approved EVENITY™ (romosozumab) for the treatment of osteoporosis in postmenopausal women at high risk of fracture. In a recent study, Saag et al. enrolled 4093 postmenopausal women with osteoporosis and a fragility fracture to compare monthly subcutaneous romosozumab (210 mg), followed by alendronate with weekly oral alendronate (70 mg), followed by alendronate for 12 months [44]. For 24 months, a 48% lower risk of new vertebral fractures was observed in the romosozumab-alendronate group (6.2%) than in the alendronateto-alendronate group (11.9%) (*p* < 0.001). Clinical fractures occurred in 9.7% of the romosozumab-alendronate group versus 13.0% of the alendronate-alendronate group, representing a 27% lower risk with romosozumab (*p* < 0.001). The risk of nonvertebral fractures was 19% lower in the romosozumab-alendronate group than in the alendronate-alendronate group (8.7% versus 10.6%; *p* = 0.04), and the risk of hip fracture was lower by 38% (2.0% versus 3.2%; *p* = 0.02) [44]. Overall adverse events and serious adverse events were comparable between the two groups. During the first year, positively adjudicated serious cardiovascular adverse events were observed more often with romosozumab than with alendronate (2.5% versus 1.9%). Adjudicated events of osteonecrosis of the jaw (one event for each group) and atypical femoral fracture (two and four events, respectively) were observed. They concluded that, in postmenopausal women with osteoporosis who were at high risk for fracture, romosozumab treatment for 12 months followed by alendronate resulted in a significantly lower risk of fracture than alendronate alone [44].

Ibandronate sodium (Boniva^®^, for treatment, 2.5 mg daily tablet, 150 mg monthly tablet, and 3 mg every three months by intravenous injection) is approved by the FDA for the treatment of postmenopausal osteoporosis. It reduces the incidence of vertebral fractures by approximately 50% over three years [29], however the drug is not efficient in reducing nonvertebral fractures.

Risedronate (Actonel^®^, for prevention and treatment, 5 mg daily tablet; 35 mg weekly tablet; 35 mg weekly tablet packaged with six tablets of 500 mg calcium carbonate; 75 mg tablets on two consecutive days every month; and 150 mg monthly tablet) is approved by the FDA for the prevention and treatment of postmenopausal osteoporosis. Over three years, it reduces the incidence of vertebral fractures by approximately 41–49% and nonvertebral fractures by approximately 36% in patients with a prior vertebral fracture [29], with significant risk reduction occurring after the first year of treatment.

Zoledronate or Zoledronic acid (ZA) (Aclasta^®^ is called Reclast^®^ in the USA. Zoledronic acid 5 mg/100 mL/vial by intravenous infusion over at least 15 min once yearly for treatment and once every two years for prevention) is a third-generation bisphosphonate approved by the FDA for the prevention and treatment of osteoporosis in postmenopausal women. In contrast to other nitrogen-containing bisphosphonates, ZA has two nitrogen atoms contained in a heterocyclic imidazole ring. Over three years, it reduces the incidence of vertebral fractures by approximately 70% (with a significant reduction in the first year), hip fractures by approximately 41%, and nonvertebral fractures by approximately 25% [29,45].

Using high-resolution Fourier-transform infrared spectroscopy and small/wide-angle X-ray scattering, Mathavan et al. attempted to address the following questions: 1. Do the molecular composition and the nano-structure in the newly regenerated bone differ between healthy and osteoporotic environments? 2. How do pharmacological treatments, such as bone morphogenetic protein 7 (BMP-7), alone or synergistically combined with ZA, alter callus composition and nano-structure in such environments [46]? According to the findings of compositional and nano-structural characterizations of newly formed bones in an open-osteotomy rat model, the healing response in untreated healthy and ovariectomy-induced osteoporotic environments was fundamentally the same [46]. However, the BMP-7 induced osteogenic response resulted in greater heterogeneity in the nano-structural crystal dimensions, and this effect was more pronounced with osteoporosis. ZA alleviated the effects of the upregulated catabolism induced by both BMP-7 and an osteoporotic bone environment [46]. Their findings contribute to the current understanding of how the repair processes in healthy and osteoporotic bone differ in both untreated and treated contexts.

Bisphosphonates prevent fractures in patients with osteoporosis, however their efficacy in postmenopausal women with osteopenia (a high-risk population for future fractures) remains unknown. Reid et al. conducted a six year, double-blind trial involving 2000 women with osteopenia (a T score of −1.0 to −2.5). Participants (65 years of age or older) were randomly assigned to receive four infusions of either 5 mg of zoledronate (zoledronate group) or normal saline (placebo group) at 18 month intervals. The primary end point was the time to the first occurrence of a nonvertebral or vertebral fragility fracture [47]. In their study, the T score at the femoral neck was −1.6 ± 0.5, and the median 10 year risk of hip fracture was 2.3%. A fragility fracture occurred in 190 women in the placebo group and in 122 women in the zoledronate group (HR: 0.63; 95% CI: 0.50–0.79; *p* < 0.001). As compared with the placebo group, women who received zoledronate had a lower risk of nonvertebral fragility fractures (HR: 0.66; *p* = 0.001), symptomatic fractures (HR: 0.73; *p* = 0.003), vertebral fractures (HR 0.45; *p* = 0.002), and height loss (*p* < 0.001) [47]. They concluded that the risk of nonvertebral or vertebral fragility fractures was significantly lower in women with osteopenia who received zoledronate [47].

In Japan, Shiraki et al. have investigated the pharmacokinetics and assessed the safety of and changes in bone metabolism associated with a single injection (4 or 5 mg) of zoledronic acid treatment in primary osteoporosis [48]. The levels of bone resorption and formation markers decreased from day 15 and from three months after administration respectively. No serious adverse events were reported. There was no large difference between the 4 and 5 mg groups in terms of pharmacokinetics, changes in the levels of bone turnover markers, and safety profiles. Their study demonstrated the acceptable pharmacokinetics and changes in bone metabolism associated with zoledronic acid treatment, and both the 4 mg dose and the 5 mg dose demonstrated acceptable safety and sustained antiresorptive effects for the duration of the study [48].

Few studies have evaluated the nature of osteoporosis-associated low back pain in a clinical situation. Fujimoto et al. have researched the nature of osteoporosis-associated low back pain without fracture, and the analgesic effect of minodronic acid hydrate on such pain. They examined 113 patients with osteoporotic low back pain and no lower extremity symptoms [49]. The following factors were evaluated before and after minodronic acid hydrate administration: questionnaires, pain rating scale, bone mineral density (BMD) of the lumbar spine, and the serum concentration of tartrate-resistant acid phosphatase 5b (TRACP-5b) as a bone metabolism marker [49]. TRACP-5b has been proven as a useful marker for bone resorption during the treatment of osteoporosis with biphosphonates [50]. In this study, osteoporosis-associated low back pain consisted of 85% nociceptive pain and 15% neuropathic or mixed pain. They found the average pain scores at rest decreased significantly two months after treatment (*p* < 0.01), while those in motion decreased significantly one month after treatment (*p* < 0.04). The average lumbar spine BMD increased after treatment, but not significantly. The changes in the average serum concentration of TRACP-5b significantly decreased one month after treatment [49].

#### 3.2.3. Adverse Effects of Bisphosphonates

Side effects are similar for all types of oral bisphosphonate medications, and include gastrointestinal problems such as difficulty in swallowing, esophageal inflammation, and gastric ulcers. When taken orally, bisphosphonates are poorly absorbed and can cause esophageal irritation/gastrointestinal symptoms. Therefore, patients must fast overnight prior to ingestion. After taking these medications, patients must wait at least 30 min before eating, drinking, or taking any other medication, to ensure adequate absorption. Furthermore, they must remain upright (sitting or standing) during this interval to avoid upper gastrointestinal symptoms [37].

Regarding intravenous bisphosphonate therapy, questions have been raised about its association with possible side effects, such as musculoskeletal pain, atrial fibrillation, esophageal cancer, and ONJ, which occur in cancer patients receiving intravenous bisphosphonate therapy. The side effects appear to be rare and may not be causally related [37].

The major problem with ZA is the postinfusion syndrome, which is common with all intravenous bisphosphonates following the first infusion. The syndrome is usually mild and can be alleviated by acetaminophen [51]. Prior to infusion, patients may be treated with acetaminophen to reduce the risk of an acute phase reaction (arthralgia, headache, myalgia, and fever). Such symptoms occurred in 32% of patients after the first dose, 7% after the second dose, and 3% after the third dose [51]. Moreover, unexpected atrial fibrillation is described as a severe adverse event which occurred in the ZA-treated group. The ZA-treated group had a higher risk of developing atrial fibrillation (1.3%) than the placebo group (0.4%). The effect of other bisphosphonates on the incidence of atrial fibrillation is uncertain [29].

Reports are available on ONJ and visual disturbances being presented by patients with cancer who underwent intravenous bisphosphonate treatment. ONJ is frequently presented as a “classical complication” of bisphosphonate treatment. Therefore, it generates anxiety in osteoporotic patients and confusion in their physicians. The level of risk for osteonecrosis in osteoporotic patients treated with bisphosphonates is unknown, but appears to be extremely small for at least up to five years [52]. According to a recent systematic review of bisphosphonate-associated ONJ in patients with cancer, ONJ is rare, with an estimated incidence of <1 case per 100,000 people—years of exposure [53]. Moreover, the pathogenesis of bisphosphonate-related ONJ remains an enigma [54].

Unusual and atypical mid-shaft long bone fractures have been reported in some patients receiving bisphosphonates, mainly alendronate, for the treatment of osteoporosis [55,56]. This could be due to the long-term oversuppression of bone turnover, which leads to impaired bone remodeling [37], the accumulation of microdamage, and the development of hypermineralized bone, however, this explanation remains to be confirmed. A number of case reports have described low-energy subtrochanteric femoral fractures and pelvic insufficiency fractures in patients on long-term bisphosphonate therapy. Bone biopsies in such patients often exhibit severely suppressed bone turnover. However, Abrahamsen et al. [57] reported that these atypical fractures are more likely due to osteoporosis rather than the bisphosphonate therapy itself [37].

A report from the American Society for Bone and Mineral Research (ASBMR) suggested that the incidence of atypical femoral fractures associated with bisphosphonate therapy appeared to be very low, particularly compared with the number of vertebral, hip, and other fractures prevented by bisphosphonates [58]. The ASBMR also found evidence of the relationship between long-term bisphosphonate use (usually more than three years, median seven years) and a specific type of subtrochanteric and femoral shaft fracture. Moreover, the apparent increased risk for atypical femoral fractures in patients receiving glucocorticoids is a concern, because bisphosphonates are the mainstay for the prevention of glucocorticoid-induced osteoporotic fractures [59].

#### 3.2.4. SERMs

Raloxifene (Evista^®^) is the only SERM approved by the FDA for both the prevention and treatment of osteoporosis in postmenopausal women. SERMs are non-steroidal compounds with tissue-specific actions. SERMs induce a different response of the ER than estradiol [59] and enhance osteoclast apoptosis [60]. In postmenopausal women, raloxifene reduces the risk of vertebral fractures by approximately 30% in patients with a prior vertebral fracture and approximately 55% in patients without a prior vertebral fracture over three years. However, it does not protect against nonvertebral or hip fractures [29,61].

Raloxifene increases the risk of deep vein thrombosis to a degree similar to that observed with estrogen, however it does not reduce the risk of coronary heart disease. It also increases hot flashes [29]. In the Multiple Outcomes of Raloxifene Evaluation (MORE) study, Ettinger et al. [62] investigated the effect of raloxifene therapy on the risk of vertebral and nonvertebral fractures in 7705 postmenopausal women with osteoporosis. They found that the risk of vertebral fractures was lower in the study groups receiving raloxifene than in the placebo group. The risk of nonvertebral fractures was similar for the two groups. Compared with the placebo group, raloxifene increased BMD in the femoral neck by 2.1–2.4% and in the spine by 2.6–2.7% (*p* < 0.001). Moreover, women treated with raloxifene had an increased risk of venous thromboembolism compared with the placebo group (RR: 3.1; 95% CI: 1.5–6.2), however, raloxifene was associated with a decreased incidence of breast cancer. Reanalyzing the MORE study, Kanis et al. [63] concluded that treatment with raloxifene significantly decreased the risk of new vertebral fractures (47%) and new clinical vertebral fractures (75%) in postmenopausal women with osteopenia at the total hip, but without vertebral fracture.

In conclusion, raloxifene at a daily dose of 60 mg can significantly decrease the risk of vertebral fractures in postmenopausal women with osteoporosis. Data on nonvertebral fractures are only positive for the post-hoc analyses of a subgroup of patients with prevalent vertebral fractures. Another clinical advantage for raloxifene is a reduced risk of invasive breast cancer, chiefly of ER-positive invasive breast cancers, which is similar to the advantage conferred by tamoxifen. However, raloxifene does not confer any cardiovascular prevention. Conversely, it causes a small but significant increase in the risk of fatal strokes and venous thromboembolism.

#### 3.2.5. PTH Peptide: Teriparatide

Teriparatide (Forteo^®^), which is a fragment of full-length PTH (1–34), is approved by the FDA for the treatment of osteoporosis in postmenopausal women and men at high risk for fracture. Teriparatide is also approved for treatment in men and women at high risk of fracture and having osteoporosis associated with sustained systemic glucocorticoid therapy. PTH is also used to increase bone mass in men with primary or hypogonadal osteoporosis, who are at high risk of fracture [29]. On the other hand, Abaloparatide (brand name Tymlos; formerly BA058) is a parathyroid hormone-related protein (PTHrP) analog drug used to treat osteoporosis. Like the related drug teriparatide, and unlike bisphosphonates, it is an anabolic (i.e., bone growing) agent. On 28 April 2017, it was approved by FDA to treat postmenopausal osteoporosis.

The use of PTH in the treatment of osteoporosis is based on the observation that intermittent exposure to low-dose PTH promotes bone anabolism, which is in contrast to the catabolic effects on the cortical bone resulting from continuous exposure to supra-physiological levels of PTH from either endogenous or exogenous origin. PTH releases calcium and phosphorus by stimulating the osteoclastic activity in the bone. Thus, the continuous secretion of excessive PTH causes bone resorption. However, when PTH is given at a low intermittent dosage, its anabolic properties are much more clearly seen [64]. PTH, which is administered by daily subcutaneous injections, stimulates bone formation. The anabolic actions of intermittently administered peptides from the PTH family involve the augmentation of the number of osteoblasts through the stimulation of cell replication, inhibition of osteoblast apoptosis, and probably the stimulation of osteoblast activity. The underlying molecular mechanisms are still poorly understood, but appear to include both direct actions on osteoblastic cells as well as indirect effects, such as the stimulation of IGF-1 production and the down-regulation of sclerostin, which is a physiological antagonist of the crucial anabolic Wnt-β-catenin pathway. The anabolic effects of PTH and related peptides are likely to be more pronounced on cancellous bone than on cortical bone [65].

The clinical use of PTH, such as teriparatide, in a dose of 20 μg daily has been shown to decrease the risk of vertebral fractures by 65% and nonvertebral fractures by 53% in patients with osteoporosis after an average of 18 months of therapy. Teriparatide is well tolerated, however some patients experience leg cramps and dizziness. In a study, 9% of the women in the 20 μg teriparatide group had dizziness and 3% had leg cramps, whereas 6% of the women in the placebo group had dizziness and 1% had leg cramps [31]. Because teriparatide causes an increase in the incidence of osteosarcoma in rats from an aspect of toxicology rather than pharmacology, patients with an increased risk of osteosarcoma (e.g., patients with Paget’s disease of the bone) and those with prior radiation therapy of the skeleton, bone metastases, hypercalcemia, or a history of skeletal malignancy should not receive teriparatide therapy. The safety and efficacy of teriparatide have not been established beyond two years of treatment. Therefore, teriparatide is used for a maximum of two years, following which the patient can be treated with an anti-resorptive agent, usually a bisphosphonate, to maintain or further increase BMD [38].

BMD decreases when teriparatide and denosumab are discontinued without follow-up antiresorptive therapy. Leder et al. conducted a study to compare rates of bone loss in postmenopausal women who received two years of either teriparatide, denosumab, or both medications, followed by two years of the alternative therapy to that in women who discontinue these drugs, followed by no antiresorptive therapy [66]. In the women not receiving follow-up therapy, femoral neck, total hip, and spine BMD decreased by 4.2%, 4.5%, and 10.0%, respectively, while BMD was maintained in those who received follow-up antiresorptive drugs (femoral neck, total hip, and spine BMD changes of −0.6%, −0.8%, and −1.2%, respectively, *p* < 0.001) [66]. In summary, the large teriparatide and denosumab-induced gains in BMD achieved with four years of intensive therapy were maintained in patients who received prompt antiresorptive therapy, but not in those left untreated. These results demonstrate the negative consequences of delaying consolidation therapy in women treated with these drugs and highlight the importance of timely medication transitions in such patients [66].

In a study enrolling 121 osteoporotic women (mean age 82.4 years), Nakatoh studied the changes in BMD and the turnover rate after 24 months of daily teriparatide (20 μg/day) administration, followed by randomly administered minodronate (50 mg/28 days), raloxifene (60 mg/day), or eldecalcitol (0.75 μg/day) [67]. In the minodronate group, BMD increased significantly from week 0 to weeks 24 and 48. The turnover rate was significantly reduced at week 12, and remained so over the entire course in all three groups. The speed of change of turnover rate was greatest in the minodronate group. In conclusion, the BMD-increasing effect was greatest with minodronate administration after teriparatide discontinuation [67].

### 3.3. Nutrition—Intake of Calcium and Vitamin D

Calcium and vitamin D play a major role in preventing and managing osteopenia and osteoporosis. Lifetime adequate calcium intake is crucial to obtain peak bone mass, which occurs between 20 and 30 years of age. According to the WHO, recommended calcium allowances based on North American and western European data and the current recommendations of the European Union, Australia, Canada/United States, and the United Kingdom, the adequate intake of calcium in children aged 7–9 is approximately 400–700 mg per day, for pubertal and adolescent boys and girls, the adequate intake is approximately 800–1300 mg per day, and for men aged 19–65 years and women aged 19 years to menopause, the adequate intake is approximately 800–1000 mg per day. The National Osteoporosis Foundation suggests that men older than 65 years and postmenopausal women should consume at least 1200 mg of elemental calcium per day. As a treatment of osteoporosis, the Committee on Medical Aspects of Food and Nutrition Policy recommends taking more than 700 mg of calcium per day to maintain bone health. Consuming 1000 mg of calcium per day reduces hip fractures by 24% [68]. Increasing dietary calcium should be the first consideration. When adequate dietary calcium intake cannot be achieved, calcium supplements could be used. Calcium carbonate is bioavailable when taken with a meal. Calcium citrate is recommended for patients with a history of renal stones [38].

Vitamin D is crucial for maintaining serum calcium and phosphate concentration and is essential for the development and maintenance of bone strength. There are limited dietary sources of vitamin D, such as salt-water oily fish and liver, egg yolk, margarine, some yogurts, cheeses, cereals, and vitamin D-fortified milk and orange juice. The major source of vitamin D_3_ for most humans is the synthesis in the skin under the influence of UV light. The prevalence of vitamin D deficiency is reported to be higher in older people, probably because of insufficient skin exposure to sunlight and a decreased efficacy of vitamin D synthesis. Indoor styles of living and clothing result in low sun exposure. UV irradiation (half the minimal erythematous dose) on 1000 cm^2^ skin of the back of older patients three times per week was as effective as the oral intake of 400 IU vitamin D [69]. A vitamin D supplement of 800–1000 IU per day for adults over 50 years of age is recommended by the National Osteoporosis Foundation, especially for housebound individuals. The desired level of an adult’s serum 25(OH)D concentration is 30 ng/mL (75 nmol/L) or higher. Recent evidence reveals that higher intakes of vitamin D are safe. Vitamin D intoxication is one of the rarest reported medical conditions and is usually observed when taking >10,000 IU of vitamin D per day for more than five months [38,70]. The 25(OH) D concentration of patients at risk for hypercalciuria and hypercalcemia should be frequently monitored [71].

The consequences of vitamin D deficiency include mineralization defects and lower BMD, causing fractures. Extra-skeletal consequences may be muscle weakness, falls, and acute respiratory infection [72]. The European Calcified Tissue Society advises to improve vitamin D status by food fortification and the use of vitamin D supplements in risk groups. Fortification of foods by adding vitamin D to dairy products, bread, and cereals can improve the vitamin D status of the whole population, but quality assurance monitoring is needed to prevent intoxication. Specific risk groups, such as infants and children up to three years, pregnant women, older persons, and non-western immigrants, should routinely receive vitamin D supplements [72].

Liao et al. conducted a study to investigate 25-hydroxyvitamin D [25(OH) D] improvement and calcium-phosphate metabolism in Chinese osteoporotic patients who were postmenopausal and treated with 70 mg of alendronate sodium and 5600 IU of vitamin D3. During the 12 months, 219 women were treated with monthly alendronate/vitamin D3 (*n* = 111) or calcitriol (*n* = 108) 89P. The safety outcome measures included serum calcium and phosphate and 24 h urine calcium [73]. Absolute change in mean serum 25(OH) D level was the greatest in Vitamin D-deficient patients and least in Vitamin D-sufficient patients at months 6 and 12 (both *p* < 0.01). Serum calcium level remained significantly lower in the alendronate/vitamin D3 group than in the calcitriol group throughout the 12 months. Mean 24 h urine calcium slightly increased in the alendronate/vitamin D3 group and significantly increased in the calcitriol treatment group (+1.1 and +0.9 mmol/L at months 6 and 12; both *p* < 0.05). Calcitriol treatment was associated with more frequent hypercalciuria at month 6 (9.4% versus 18.5%, *p* = 0.05), but not at month 12 (12.3% versus 13.0%) [73]. They concluded that baseline Vitamin D status predicted 25(OH) D improvement in patients with 12 months of alendronate/vitamin D3 treatment. The daily use of calcitriol was associated with more frequent hypercalciuria at month 6, compared to alendronate/vitamin D3 treatment, necessitating the safety re-evaluation of calcitriol at a higher dosage [73].

## 4. Conclusions

In summary, all current osteoporosis drugs have considerable side effects or lack efficacy, and an ideal osteoporosis therapy has not yet been developed. Although complex, the genetics, pathogenesis, and various molecular pathways of osteoporosis are slowly being put together. Knowledge of these targets can aid us in developing new drugs in the future. For example, monoclonal antibodies that target RANKL are currently being developed [1].

Effective fracture prevention by reducing the loss of bone mass is the primary goal of physicians providing treatment to people with osteoporosis. Other than lifestyle changes, nutrition changes, and fall prevention, therapeutic choices, including pharmacologic agents, exercise, and physical therapy modalities can be considered to treat osteoporosis or prevent further osteoporotic fractures. Each choice or combination of choices can offer benefits to people with osteoporosis. A treatment plan should be selected after a detailed discussion with the osteoporosis patient. This review provides the current opinions on osteoporosis and its medical treatment. However, many issues regarding osteoporosis and its treatment remain unknown and controversial, and these issues warrant future investigation.

## Abbreviations

27HC	27-hydroxycholesterol
ASBMR	American Society for Bone and Mineral Research
bHLH	basic helix-loop-helix
BMD	bone mineral density
BMP2	bone morphogenetic protein 2
BMP-7	bone morphogenetic protein 7
BMSC	bone marrow stromal cell
CACNG1	voltage-dependent calcium channel γ1
CaMKII	calcium calmodulin-mediated kinase II
CCR3	C-C motif chemokine receptor 3
CNV	copy number variation
COL1A1	collagen, type I, alpha 1
CpG	5′—C—Phosphate—G—3
DEG	differentially expressed gene
Dkk1	Dickkopf-1
DXA	dual-energy X-ray absorptiometry
E2	estradiol
ER	estrogen receptor
ERα	estrogen receptor alpha
ERβ	estrogen receptor beta
ESR1	estrogen receptor 1
ET	estrogen therapy
FDA	US Food and Drug Administration
GCR	glucocorticoid receptor
GI	gastrointestinal
GWAS	genome-wide association studies
HDC	histidine decarboxylase
HRT	hormone replacement therapy
Id4	Inhibitor of differentiation 4
IGF-1	Insulin-like growth factor 1
JNK	c-Jun N-terminal kinase
LRP5	Low-density lipoprotein receptor-related protein 5
M-CSF	macrophage colony stimulating factor
MSC	mesenchymal stem cell
mTECs	medullary thymic epithelial cells
NFAT	nuclear factor of activated T cells
ONJ	osteonecrosis of the jaw
OPG	osteoprotegerin
PEG	polyethylene glycol
Pi	phosphate
PO	postmenopausal osteoporosis
PPARγ	peroxisome proliferator activator gamma
PPI	protein-protein interaction
PTH	parathyroid hormone
QTL	quantitative trait locus
RANK	receptor activator of nuclear factor kappa-B
RANKL	receptor activator of nuclear factor kappa-B ligand
RCT	randomized controlled trial
Runx2	runt-related transcription factor 2
RXR	retinoid X receptor
SAMP6	Senescence-accelerated mouse prone 6
SERMs	Selective Estrogen Receptor Modulators
SFRP	secreted frizzled-related protein
SFRP4	secreted frizzled-related protein 4
*SGMS2*	sphingomyelin synthase 2 gene
SMAD4	Mothers against decapentaplegic homolog 4
SNP	single-nucleotide polymorphism
TF	transcription factor
TRACP-5b	tartrate-resistant acid phosphatase 5b
TSECs	Tissue Selective Estrogen Receptor Complexes
UGT2B17	UDP Glucuronosyl transferase Family 2 Member B17
VDR	vitamin D receptor
WHI	Women’s Health Initiative
WHO	World Health Organization
Wnt	Wingless/Integrated
ZA	Zoledronic acid
